# Current practice of biologic augmentation techniques to enhance the healing of meniscal repairs: A collaborative survey within the Meniscus International Network (MenIN) Study Group

**DOI:** 10.1002/ksa.12685

**Published:** 2025-05-07

**Authors:** James Robinson, Iain R. Murray, Gilbert Moatshe, Jorge Chahla, Luke V. Tollefson, David A. Parker, Filippo Familiari, Robert F. LaPrade, Nicholas N. DePhillipo, the Meniscus International Network (MenIN) Study Group

**Affiliations:** ^1^ Knee Specialists Bristol UK; ^2^ Edinburgh Orthopaedics University of Edinburgh Edinburgh UK; ^3^ Oslo Sport Trauma Research Center Norwegian School of Sports Science Oslo Norway; ^4^ Orthopaedic Clinic Oslo University Hospital Ullevål Oslo Norway; ^5^ Department of Orthopaedic Surgery Rush University Medical Center Chicago Illinois USA; ^6^ Midwest Orthopaedics at Rush Chicago Illinois USA; ^7^ Twin Cities Orthopedics Edina Minnesota USA; ^8^ Sydney Orthopaedic Research Institute Sydney New South Wales Australia; ^9^ Department of Orthopaedic and Trauma Surgery Magna Graecia University Catanzaro Italy; ^10^ Research Center on Musculoskeletal Health (MusculoSkeletalHealth@UMG) Magna Graecia University Catanzaro Italy; ^11^ Department of Orthopedics University of Pennsylvania Philadelphia Pennsylvania USA

**Keywords:** biologic augmentation, meniscus, Meniscus International Network (MenIN) Study Sroup, repair

## Abstract

**Purpose:**

To evaluate practices and preferences among expert sports knee surgeons regarding biologic augmentation techniques in meniscal repair.

**Methods:**

A 12‐question multiple‐choice survey was distributed to the Meniscus International Network (MenIN) Study Group. It covered biologic augmentation techniques for various meniscal tear types, both in isolation and with anterior cruciate ligament reconstruction (ACLR). Eight options were assessed: no augmentation, trephination, rasping, marrow venting, fibrin clot, platelet‐rich plasma (PRP), bone marrow aspirate concentrate (BMAC) and meniscal wrapping. Surgeons could select multiple techniques per scenario.

**Results:**

Forty‐two surgeons participated: 42% from Europe, 18% from North America, 10% from Latin America, 21% from Asia and 9% from Africa/Oceania. For isolated meniscal tears (excluding meniscal root tears), 90% of surgeons used at least one biologic augmentation technique. For meniscal tears associated with ACLR, 66% of surgeons used at least one biologic augmentation technique. The most utilized techniques were rasping (19%–69%), trephination (7%–43%), and marrow venting (0%–74%). PRP (2%–19%), BMAC (0%–14%) and meniscal wrapping (0%–10%) were least used. Biologic augmentation was most frequent for isolated radial (93%), isolated bucket‐handle (86%), isolated vertical (86%) and isolated horizontal tears (98% for younger patients, 86% for degenerative tears). ACLR‐associated repairs had lower augmentation rates, and meniscal root tears showed the highest percentage of non‐augmented repairs. Over 50% of surgeons use a single augmentation technique, while 20% use two techniques depending on tear type. Overall, 33.3% (*n* = 14) of surgeons reported utilizing PRP and/or BMAC for meniscal repair augmentation, with the highest use observed in South America (12%) based on geographic usage.

**Conclusions:**

This survey provides insights into current meniscal repair practices among expert orthopaedic sports medicine surgeons. The findings reveal variability in approaches based on tear patterns and associated procedures, with a general preference for simpler mechanical augmentation techniques over more advanced biologics. For isolated meniscal tears (excluding meniscal root tears), 90% of surgeons in this cohort report using one or more biological augmentation techniques.

**Level of Evidence:**

Level V, expert opinion.

**Abbreviations**
ACLR: anterior cruciate ligament reconstruction
BMAC: bone marrow aspirate concentrate
ISAKOS: International Society of Arthroscopy, Knee Surgery and Orthopaedic Sports Medicine
MenIN: Meniscus International Network
PRP: platelet‐rich plasma
VEGF: vascular endothelial growth factor

## INTRODUCTION

The menisci play a critical role in maintaining knee joint health, yet their limited healing potential presents a significant challenge. This has sparked exploration into biological augmentation techniques aimed at stimulating tissue regeneration, promoting healing, and improving outcomes of meniscal repair, particularly in patients with tears in less vascularised areas of the meniscus [3, 28]. Various techniques have been previously described and are in current use clinically, including trephination, rasping, marrow venting, fibrin clot, platelet‐rich plasma (PRP), bone marrow aspirate concentrate (BMAC) and meniscal wrapping [1].

Meniscal trephination involves creating small perforations in the meniscal rim to promote bleeding and stimulate healing [36]. Rasping involves abrading the surface of the meniscus and surrounding synovium to promote a healing response [30]. The use of a fibrin clot as a biological scaffold involves preparing and placing a clot at the site of the meniscal tear to provide a matrix for cell migration and tissue formation [2]. Meniscal wrapping involves the application of a biological scaffold or membrane, often derived from collagen or synthetic materials, around the meniscus to enhance the local healing environment, promote healing and provide structural support for the healing meniscus [29, 32]. PRP uses concentrated platelets from a patient's own blood and BMAC, cells from bone marrow, which are rich in growth factors that may accelerate tissue repair [22, 34, 37].

While studies have shown promising results in improving the meniscal healing process in some cases of meniscal repair, there is no clear consensus on the best approach for meniscal repair augmentation and studies comparing these methods are limited [1, 4]. Similarly, the influence of tear location on the success of biological augmentation techniques is not fully understood. Therefore, the purpose of this study was to evaluate current practices and preferences among expert sports knee surgeons regarding the use of biologic augmentation techniques in meniscal repair. By collecting data on clinical practices and preferences, this survey of the Meniscus International Study Group (MenIN) members was designed to identify trends in the application of meniscal augmentation techniques to help clarify which techniques are currently perceived to be most effective in enhancing meniscal healing and where additional research is needed. This collective knowledge will contribute to developing evidence‐based guidelines and best practices for improving outcomes in meniscal repair.

## METHODS

This study was performed by the MenIN Study Group which constitutes an international research consortium of experienced sports knee surgeons and researchers, with a dedicated focus on advancing the understanding and treatment of meniscus‐related knee pathologies, aiming to combine experience and data to facilitate the formulation of recommendations for the broader sports trauma community. This includes conducting collaborative research, developing consensus statements, and establishing standardized classification systems for meniscal conditions [13].

A 12‐question multiple‐choice, online survey was distributed via email to the 69 members of the MenIN Study Group. It was designed to address the use of different biologic augmentation techniques for the repair of specific meniscal tear types, both in isolation and when associated with anterior cruciate ligament reconstruction (ACLR). There were 12 different types of meniscal tear/repair scenarios presented with eight different multiple‐choice options regarding current use of biologic augmentation technique: ‘None’, ‘Trephination’, ‘Rasping’, ‘Marrow Venting’, ‘Fibrin Clot’, ‘PRP’, ‘BMAC’ and ‘Meniscal Wrapping’. Participants were instructed to ‘Select All That Apply’ for each clinical scenario. A free‐text explanatory section was also provided. Given the survey's structure permitting respondents to select multiple answers per clinical scenario, cumulative response frequencies occasionally exceeded 100%, reflecting overlapping data. To mitigate potential response errors and ensure clarity in depicting recommended biologic augmentation practices, instances where respondents selected both ‘None’ and one or more biologic augmentation technique options for the same scenario were adjusted. The ‘None’ responses were excluded to enhance the fidelity of the data, reinforcing this categorization as chosen only when alternative biologic augmentations were not selected. All survey participants had the opportunity to decline the questionnaire. Inclusion criteria included only those orthopaedic surgeons who currently performed meniscal repair surgery. Exclusion criteria were those researchers who do not perform meniscal repair surgery or surgeons choosing to opt out of the survey. Since no patient data was included, this study was determined to be IRB‐exempt.

### Statistical analysis

Data were collected regarding surgeon preferences, surgeon geographic location, and frequencies of biologic augmentation techniques used at the time of meniscal repair. Descriptive statistics were used to summarize the frequency and distribution of biologic augmentation techniques across different meniscal tear patterns and repair scenarios. Categorical data were analyzed using percentages and proportions to determine the prevalence of each augmentation method within specific clinical contexts. Given that participants could select multiple augmentation techniques for each scenario, response frequencies exceeded 100%, requiring an approach that accounted for overlapping selections. Subgroup analyses were performed to assess differences in biologic augmentation techniques used based on different meniscal tear patterns, meniscal tears repaired with concomitant ACLR, and the number of different biologic augmentation techniques used dependent on meniscal repair scenarios.

## RESULTS

**Figure 1:**
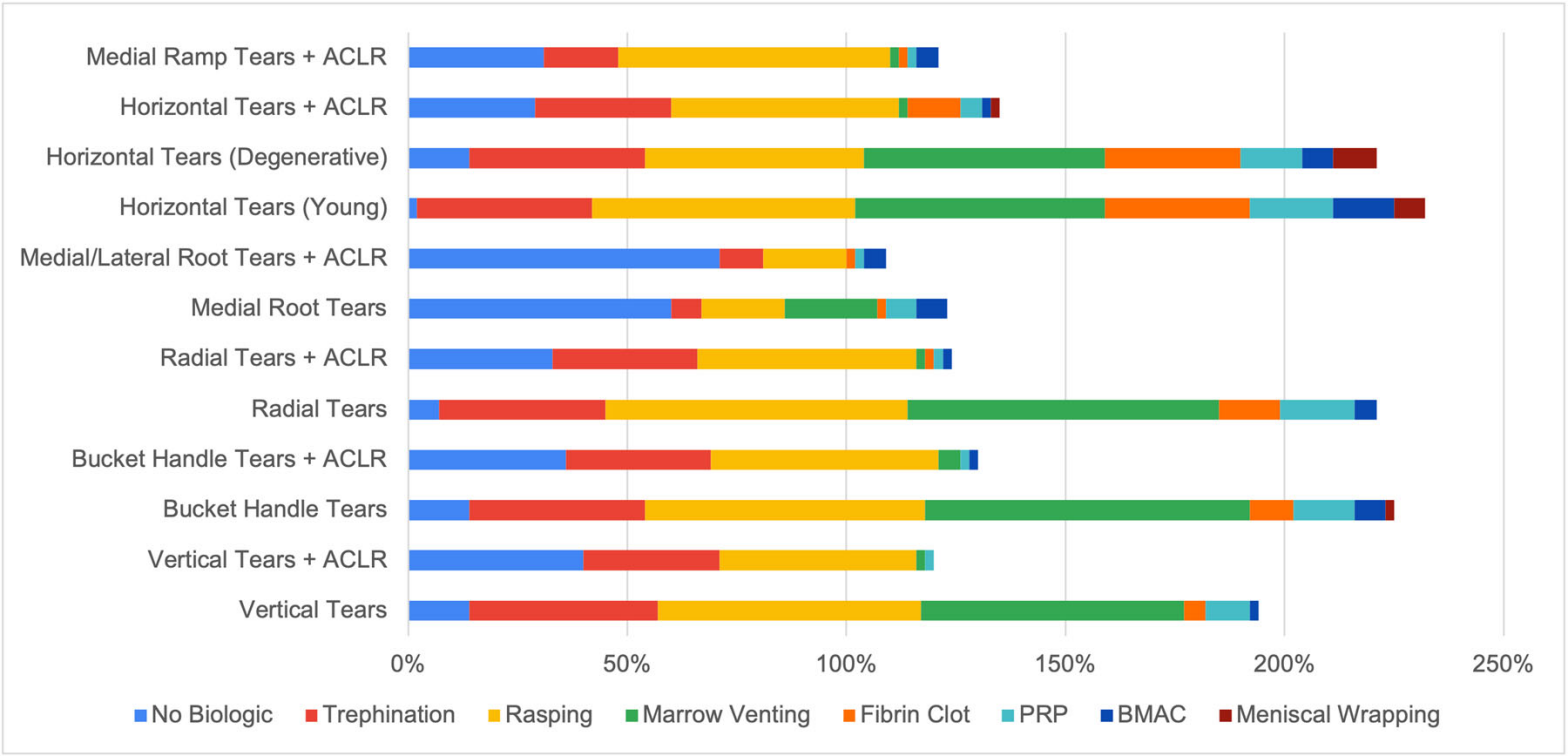
Bar chart representing the utilization of biologic augmentation techniques based on cumulative frequencies across all meniscal tear patterns and repair scenarios. ACLR, anterior cruciate ligament reconstruction; BMAC, bone marrow aspirate concentration; PRP, platelet‐rich plasma.

Of the 69 members, a total of 42 surgeons completed the survey: 42% from Europe, 18% from North America, 10% from Latin America, 21% from Asia and 9% from Africa and Oceania. Across all 12 meniscal tear patterns and repair scenarios, the utilization of biologic augmentation techniques varied. The frequency of using no biologic augmentation ranged from 10% to 74%. For isolated meniscal tears (excluding meniscal root tears), 90% of surgeons used at least one biologic augmentation technique. For meniscal tears associated with ACLR, 66% of surgeons used at least one biologic augmentation technique. The results of all biologic augmentation techniques reported in current clinical practice by the surgeons in this study are presented in Table 1 and Figure 1.

**Table 1:** Total frequencies (expressed as a percentage) of all biologic augmentation techniques reported by surgeons for different meniscal tear patterns with or without concomitant anterior cruciate ligament reconstruction (ACLR) at the time of meniscal repair (*n* = 42).

Meniscal tear pattern/clinical repair scenario	No biologic technique used	Trephination	Rasping	Marrow venting	Fibrin clot	PRP	BMAC	Meniscal wrapping
Vertical tears	14%	43%	60%	60%	5%	10%	2%	0%
Vertical tears + ACLR	40%	31%	45%	2%	0%	2%	0%	0%
Bucket handle tears	14%	40%	64%	74%	10%	14%	7%	2%
Bucket handle tears + ACLR	36%	33%	52%	5%	0%	2%	2%	0%
Radial tears	7%	38%	69%	71%	14%	17%	5%	0%
Radial tears + ACLR	33%	33%	50%	2%	2%	2%	2%	0%
Medial root tears	60%	7%	19%	21%	2%	7%	7%	0%
Medial/lateral root tears + ACLR	71%	10%	19%	0%	2%	2%	5%	0%
Horizontal tears (Young)	2%	40%	60%	57%	33%	19%	14%	7%
Horizontal tears (degenerative)	14%	40%	50%	55%	31%	14%	7%	10%
Horizontal tears + ACLR	29%	31%	52%	2%	12%	5%	2%	2%
Medial ramp tears + ACLR	31%	17%	62%	2%	2%	2%	5%	0%

Abbreviations: BMAC, bone marrow aspirate concentration; PRP, platelet‐rich plasma.

### Utilization of biologic augmentation techniques

**Figure 2:**
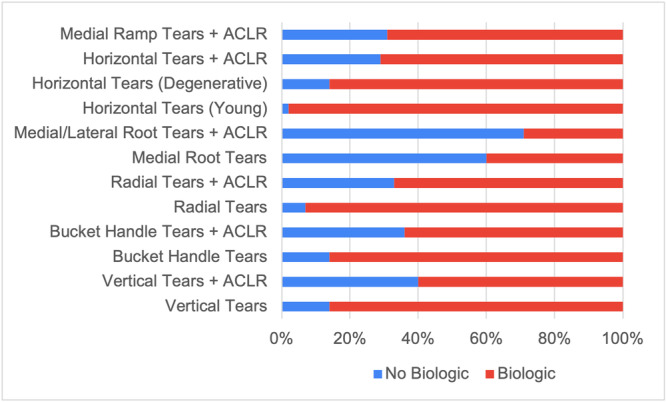
Bar chart representing the utilization of biologic augmentation compared to no biologic augmentation for each meniscal tear pattern and repair scenario. ACLR, anterior cruciate ligament reconstruction.

Meniscal rasping was reported as the most frequently used biologic augmentation technique across all scenarios, followed by trephination and marrow venting. Meniscal wrapping, BMAC and PRP were the least utilized techniques overall. The highest frequencies of biologic augmentation techniques were reported for isolated bucket‐handle tears (86%), isolated radial tears (93%), isolated vertical tears (86%), isolated horizontal tears in younger patients (98%) and isolated degenerative horizontal tears (86%). No biologic augmentation technique usage was highest for medial/lateral root tears with ACLR (71%), isolated medial root tears (64%) and vertical tears with ACLR (40%) (Figure 2).

**Figure 3:**
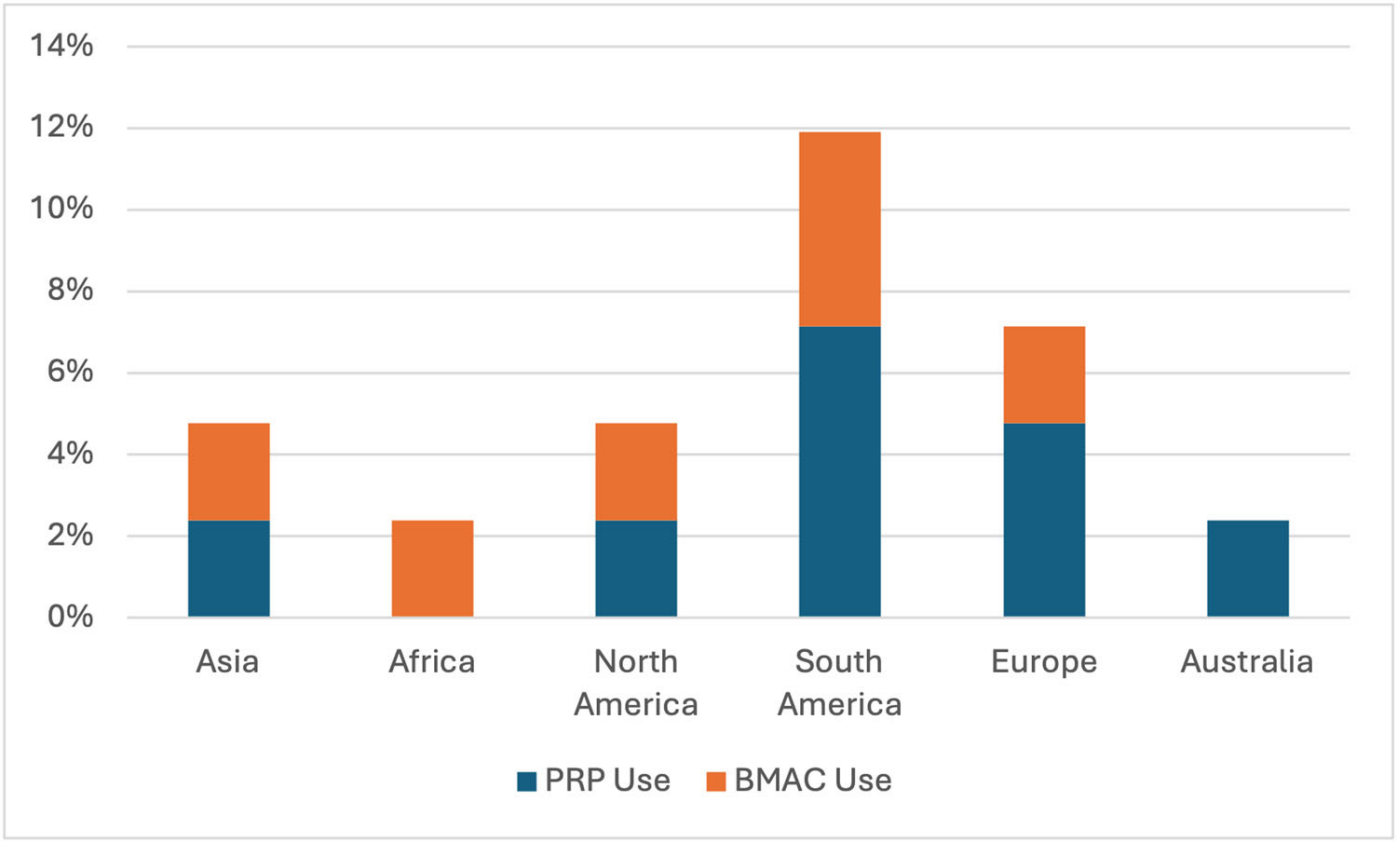
Use of platelet‐rich plasma (PRP) and bone marrow aspirate concentrate (BMAC) for biologic augmentation of meniscal repairs by geographic region. One‐third of all surgeons in this study reported use of PRP and/or BMAC for meniscal repair augmentation (*n* = 14).

The most common reported biologic augmentation techniques for each meniscal tear pattern were as follows: rasping and marrow venting for vertical tears (60%), rasping for vertical tears with an ACLR (45%), marrow venting for bucket‐handle tears (74%), rasping for bucket‐handle tears with an ACLR (52%), marrow venting for radial tears (71%), rasping for radial tears with an ACLR (50%), marrow venting for medial root tears (21%), rasping for medial/lateral root tears with an ACLR (19%), rasping for horizontal tears in younger patients (60%), marrow venting for degenerative horizontal tears in older patients (55%), rasping for horizontal tears with an ACLR (52%) and rasping for medial ramp tears with an ACLR (62%). The use of fibrin clot (33%), PRP (19%), and BMAC (14%) augmentation were highest in horizontal tears in younger patients. Meniscal wrapping augmentation was highest in degenerative horizontal tears in older patients (10%). Overall, 33.3% (*n* = 14) of surgeons reported using PRP and/or BMAC for meniscal repair augmentation, irrespective of tear type or meniscal repair scenario. The highest use of both biologics was observed in South America (12%), followed by Europe (7%), North America (5%), Asia (5%), Africa (2%) and Australia (2%) (Figure 3).

### Number of biologic augmentation techniques used during meniscus repair

Over 50% of surgeons reported one biologic augmentation during any meniscal tear pattern and clinical repair scenario, while over 20% reported two techniques (Table 2). For vertical tears, 24% of surgeons reported one biologic, 33% reported two, and 26% reported three different biologics depending on the clinical scenario. For vertical tears with a concomitant ACLR, 40% of surgeons reported to use no biologic. For isolated bucket handle tears, 14% reported use of one biologic technique, 31% two, 29% three, 10% four, and 2% five different biologic techniques. The clinical scenarios where surgeons reported the use of five or more biologics to augment meniscal repair were horizontal tears in younger patients (5%), horizontal degenerative tears (5%), isolated radial tears (2%) and isolated bucket‐handle tears (2%).

**Table 2:** Total frequencies (expressed as a percentage) of the number of different biologic augmentation techniques used by surgeons according to meniscal tear pattern/clinical repair scenario (*n* = 42).

Meniscal tear pattern/clinical repair scenario	No Biologic	1 Biologic	2 Biologics	3 Biologics	4 Biologics	5+ Biologics
Vertical tears	14%	24%	33%	26%	2%	0%
Vertical tears + ACLR	40%	40%	17%	2%	0%	0%
Bucket handle tears	14%	14%	31%	29%	10%	2%
Bucket handle tears + ACLR	36%	38%	21%	5%	0%	0%
Radial tears	7%	21%	29%	38%	2%	2%
Radial tears + ACLR	33%	43%	21%	2%	0%	0%
Medial root tears	60%	26%	7%	5%	2%	0%
Medial/lateral root tears + ACLR	71%	21%	5%	2%	0%	0%
Horizontal tears (Young)	2%	31%	24%	24%	14%	5%
Horizontal tears (degenerative)	14%	29%	12%	31%	10%	5%
Horizontal tears + ACLR	29%	43%	24%	2%	2%	0%
Medial ramp tears + ACLR	31%	50%	17%	2%	0%	0%

Abbreviations: ACLR, anterior cruciate ligament reconstruction.

## DISCUSSION

The most important finding of this study was that simpler mechanical techniques, such as rasping, trephination, and marrow venting, were the most frequently utilized methods for biologic augmentation in meniscal repair. Regardless of meniscal tear pattern/clinical scenario, most surgeons in this cohort currently report using biological augmentation techniques to enhance meniscus repair procedures, with 90% using at least one biological augmentation technique for isolated meniscus body tears. Advanced biologics, including PRP, BMAC and meniscal wrapping, were the least commonly utilized. Surgeons reported using fewer biologic augmentation techniques when meniscal repair was performed in conjunction with an ACLR, likely due to the belief that ACLR itself provides sufficient biological stimulation for healing through the liberation of marrow elements into the joint from tunnel preparation. No biologic augmentation was highest when treating meniscal root tears (60%–71%), whereas adding a biologic augmentation was most frequently used when treating horizontal and radial tears (86%–98%). More surgeons used multiple biologic augmentation techniques compared to a single technique for isolated meniscal tears, suggesting a desire to maximize healing enhancement for more complex tears, yet with a general inclination towards simpler mechanical techniques (rasping, trephination and marrow venting) over advanced biologics.

This international survey revealed that orthopaedic surgeons currently utilize mechanical stimulation techniques more frequently than advanced biologics for augmenting meniscal repair. This preference for mechanical methods over advanced biology may stem from the simplicity and familiarity of these techniques, as well as concerns regarding the efficacy and cost‐effectiveness of autologous biologics such as PRP and BMAC [1, 6, 11]. Rasping, trephination, and marrow venting are widely used due to their ability to stimulate vascularization and fibrocartilage formation in the avascular zones of the meniscus [5, 18]. Studies have demonstrated that these mechanical methods are effective in promoting healing without significantly altering meniscal collagen architecture [17, 23, 24]. Historically, Fox et al. [14] reported a 90% success rate with trephination in peripheral meniscal tears, while Zhang and Arnold [36] observed fewer symptomatic retears when trephination was combined with suture repair compared to suture repair alone. Keller et al. [20] reported that mechanical augmentation techniques are often favoured because they are less resource‐intensive and easier to integrate into routine surgical practice compared to advanced biologics such as PRP and BMAC.

While PRP and BMAC have demonstrated potential for improving cell proliferation and extracellular matrix production in preclinical settings, their clinical efficacy remains inconclusive [7, 12, 19, 27, 31]. Zaffagnini et al. [35] reported a failure rate of 9.9% for PRP‐augmented repairs compared to 25.7% for controls. Koch et al. [21] reported significant healing improvements with BMAC in avascular meniscal tears in a preclinical animal model. A prospective case–control study reported that while BMAC provided better pain reduction and healing at early time points (6 weeks and 3 months), long‐term outcomes of meniscal repair at 1 year were comparable between BMAC‐augmented repairs and controls [26]. Dancy et al. [7] found that while BMAC or PRP augmentation reduced revision surgery rates in meniscus repairs performed with ACLR, no statistically significant difference was observed in augmented isolated meniscus repairs compared to controls. These findings suggest that the limited adoption of advanced biologics observed in this survey may be influenced by a lack of robust clinical evidence supporting their efficacy.

Cost‐effectiveness is another critical factor influencing the choice of biologic augmentation techniques. Mechanical methods like rasping and trephination are relatively inexpensive and require minimal additional resources, making them accessible even in resource‐limited settings. In contrast, advanced biologics such as PRP and BMAC involve higher upfront costs for preparation and administration. A recent economic analysis found that marrow venting procedures were more cost‐effective than PRP‐augmented repairs, with similar quality‐adjusted life years at a fraction of the cost [27]. Additionally, variability in preparation techniques for PRP and BMAC contributes to inconsistent results, further complicating their adoption in clinical practice [22]. This aligns with the current survey findings, where simpler techniques were preferred over costlier alternatives. The use of fibrin clot is another cost‐effective technique with a recent retrospective study showing its use resulted in clinical healing in up to 90% of meniscus repair cases [8, 9]. [Correction added on 23 May 2025, after first online publication: Davies et al. has been added as Reference 8. All subsequent references and citations have been renumbered in this version.] However, other studies have questioned its efficacy with a 2022 review reporting an overall success rate of 70%–75%, similar to meniscus repair without augmentation [31]. Further research is needed to assess the cost‐effectiveness of various biologic augmentation strategies to inform clinical practice and to evaluate the superiority of advanced biologic techniques to justify their routine use in augmenting meniscal repair. This current survey revealed that the choice of biologic treatments for meniscal repair is influenced by the specific tear pattern and whether the repair is performed in conjunction with an ACLR. The results highlight that biologic augmentation is most employed in tear patterns where healing is inherently challenging due to poor vascularity or complex tear morphology, such as radial and bucket‐handle tears [24, 25, 26]. Notably, surgeons reported a reduced use of biologic augmentation techniques when meniscal repair was combined with an ACLR. This trend may be attributed to the belief that an ACLR inherently provides sufficient biological stimulation for meniscal healing through mechanisms such as intra‐articular bleeding from bone tunnel drilling, which releases growth factors and mesenchymal stem cells into the joint environment [10, 12, 16]. Galleria et al. [15] found that reaming tunnels during ACLR led to significantly increased levels of vascular endothelial growth factor in the knee joint fluid compared to partial meniscectomy alone. These findings align with prior studies suggesting that ACLR‐associated meniscal repairs exhibit higher healing rates compared to isolated meniscal repairs due to this favourable biological milieu [9, 33]. Thus, the use of advanced biologics during meniscal repair with concomitant ACLR may not provide additional benefits to the patient and warrants further investigation.

### Limitations

This study is limited by its survey‐based design, which relies on self‐reported data from surgeons, introducing the potential for response bias and variability in interpreting biologic augmentation techniques. Due to the nature of the survey design which allowed respondents to ‘select all’ answers for each clinical question, response frequencies could exceed 100% with overlapping data. Additionally, the study does not include clinical outcome data, which limits the ability to assess the efficacy of these reported techniques in improving meniscal healing or correlate reported clinical practices with patient outcomes. The sample size, while composed of experienced knee surgeons, may not fully represent global practice patterns, and the study does not account for factors such as patient‐specific variables, surgical skill or institutional resources or protocols that may influence technique selection. To offset these limitations, future studies should incorporate prospective clinical trials or registry‐based data to evaluate the real‐world effectiveness of biologic augmentation techniques. Further research is needed to correlate current clinical practices with patient outcomes, helping to establish evidence‐based guidelines for biologic augmentation in meniscal repair.

## CONCLUSION

This survey provides insights into the current clinical practice patterns for meniscal repair among an expert group of international orthopaedic sports medicine surgeons who specialize in treating meniscal pathology. The results highlight the variability in approach based on tear patterns and associated procedures, as well as a general preference for simpler, mechanical augmentation techniques. For isolated meniscal tears (excluding meniscal root tears), 90% of surgeons in this cohort report using at least one biological augmentation technique in an attempt to enhance healing during meniscal repair.

## AUTHOR CONTRIBUTIONS

All authors have read and approved the final submitted manuscript. Substantial conception/design of work: James Robinson, Iain Murray, Robert F. LaPrade and Nicholas N. DePhillipo. Data collection: James Robinson, Iain Murray and Nicholas N. DePhillipo. Data analysis: James Robinson, Gilbert Moatshe and David A. Parker. Interpretation of data: Iain Murray, Jorge Chahla and Filippo Familiari. Drafting the work: James Robinson, Iain Murray and Nicholas N. DePhillipo. Critically revising the work: Gilbert Moatshe, Jorge Chahla, David A. Parker, Luke Tollefson and Robert F. LaPrade. Manuscript preparation: Filippo Familiari, James Robinson, Iain Murray and Nicholas N. DePhillipo. Approving final version for publication: All authors. Agreement for accountability of all aspects of work: All authors.

## THE MENISCUS INTERNATIONAL NETWORK (MENIN) STUDY GROUP

Corrado Bait, MD, Giuseppe Calafiore, MD, Jourdan Cancienne, MD, Pieter D'Hooghe, MD, Lars Engebretsen, MD, PhD, João Espregueira‐Mendes, MD, PhD, Scott C. Faucett, MD, MS, David Figueroa, MD, Takayuki Furumatsu, MD, PhD, Alan Getgood, MPhil, MD, FRCS(Tr&Orth), Safa Gursoy, MD, PhD, Camilo Partezani Helito, MD, PhD, Gazi Huri, MD, PhD, Hideyuki Koga, MD, PhD, Sebastian Kopf, MD, Christopher M. LaPrade, MD, Sang Hak Lee, MD, Rodrigo Maestu, MD, Joan Carles Monllau, MD, PhD, Nicolas Pujol, MD, José Leonardo Rocha de Faria, MD, MSc, Adnan Saithna, MD, FAANA, Kristian Samuelsson, MD, PhD, MSc, Roberto Simonetta, MD, Bertrand Sonnery‐Cottet, MD, PhD, Tim Spalding, FRCS(Orth), Ciara Stevenson, FRCS(Orth), Sachin Tapasvi, MBBS, MS(Ortho), DNB, FRCS(Glasgow), María Jesús Tuca, MD, René Verdonk, MD, PhD, Peter Verdonk, MD, PhD, Gustavo Vinagre, MD, PhD, Richard von Bormann, MBChB, FC Ortho (SA), Stefano Zaffagnini, MD, PhD.

## CONFLICT OF INTEREST STATEMENT

Robert F. LaPrade is a consultant for Ossur and Smith and Nephew; receives royalties from Ossur, Smith and Nephew and Elsevier; and research grants from AOSSM, AANA, Ossur, Arthrex and Smith and Nephew. The remaining authors declare no conflicts of interest.

## ETHICS STATEMENT

The ethics statement is not available.

## Data Availability

The raw data and materials supporting the findings of this study are available upon request from the corresponding author.
